# Ketogenic diet preserves muscle mass and strength in a mouse model of type 2 diabetes

**DOI:** 10.1371/journal.pone.0296651

**Published:** 2024-01-10

**Authors:** Sol Been Park, Soo Jin Yang

**Affiliations:** Department of Food and Nutrition, Seoul Women’s University, Seoul, Republic of Korea; Duke University School of Medicine, UNITED STATES

## Abstract

Diabetes is often associated with reduced muscle mass and function. The ketogenic diet (KD) may improve muscle mass and function via the induction of nutritional ketosis. To test whether the KD is able to preserve muscle mass and strength in a mouse model of type 2 diabetes (T2DM), C57BL/6J mice were assigned to lean control, diabetes control, and KD groups. The mice were fed a standard diet (10% kcal from fat) or a high-fat diet (HFD) (60% kcal from fat). The diabetic condition was induced by a single injection of streptozotocin (STZ; 100 mg/kg) and nicotinamide (NAM; 120 mg/kg) into HFD-fed mice. After 8-week HFD feeding, the KD (90% kcal from fat) was fed to the KD group for the following 6 weeks. After the 14-week experimental period, an oral glucose tolerance test and grip strength test were conducted. Type 2 diabetic condition induced by HFD feeding and STZ/NAM injection resulted in reduced muscle mass and grip strength, and smaller muscle fiber areas. The KD nutritional intervention improved these effects. Additionally, the KD altered the gene expression of nucleotide-binding and oligomerization domain-like receptor family pyrin domain-containing 3 (NLRP3) inflammasome- and endoplasmic reticulum (ER) stress-related markers in the muscles of diabetic mice. Collectively, KD improved muscle mass and function with alterations in NLRP3 inflammasome and ER stress.

## Introduction

The prevalence of diabetes in adults was about 10.5% (536.6 million) in 2021, and is expected to rise to 12.2% (783.2 million) in 2045 [1]. Diabetes is frequently accompanied by reduced muscle mass and function, and sarcopenia was more prevalent in people with diabetes than non-diabetics [2, 3]. Dietary interventions have shown promise in reducing symptoms and delaying diabetic complications, including diabetic sarcopenia [4, 5].

The low-carbohydrate and fat-rich (typically about 80~90% energy from fat) ketogenic diet (KD) induces nutritional ketosis [6], and helps reduce the frequency and extent of seizures in patients with epilepsy [7]. Moreover, KD, or a very low-calorie KD, is protective against weight gain and cancers [8–10]. The KD also exerts a glycemic control effect [11, 12], reducing the need for insulin and anti-diabetic agents. Blood ketone concentrations were mildly higher in patients with T2DM [13, 14]. However, the blood ketone concentrations were inversely associated with insulin resistance [13], implying that increased ketone production from KD ingestion might improve insulin action. The KD-mediated improvements in glycemic control may be related to the modulation of insulin signaling, inflammation and the post-translational modification through the histone beta-hydroxybutyrylation [15, 16].

Sarcopenia is prevalent in diabetes, especially in the elderly [17], and is associated with increased mortality in patients with T2DM [18]. Impaired insulin signaling is an underlying cause of sarcopenia in diabetes [19, 20]. Defective insulin activity reduces muscle protein anabolism and increases protein catabolism [19]. In addition to impaired insulin action, inflammation, endoplasmic reticulum (ER) stress, and oxidative stress may contribute to muscle deterioration [20, 21]. Excessive inflammation via nucleotide-binding and oligomerization domain-like receptor family pyrin domain-containing 3 (NLRP3) inflammasome activation and increased ER stress were reported in diabetes, which may impair insulin action [22–25]. Subsequently, inhibition of insulin-mediated anabolic processes can reduce muscle mass and impair muscle function. Considering the prevalence of sarcopenia in diabetes and its increased mortality risk, practical strategies to prevent or treat the progression of diabetic sarcopenia are needed, including nutritional intervention. However, limited data are available regarding the effectiveness of dietary interventions on diabetic sarcopenia and the underlying mechanisms. General knowledge regarding the effects of KD on muscle mass and function is limited and has focused primarily on age-related sarcopenia or athletes [26–28], not on diabetic sarcopenia.

In the current study, we hypothesized that the KD would preserve muscle mass and strength in diabetic mice (DM). To test this hypothesis, the effects of the KD on muscle mass, muscle fiber morphology, and grip strength were investigated in a mouse model of T2DM. Additionally, gene expression of NLRP3 inflammasome- and ER-stress-related targets was analyzed to determine whether the KD alters NLRP3 inflammasome and ER stress in the muscles of DM. The current study demonstrated the effects of the KD on preserving muscle mass, muscle fiber size, and muscle function.

## Materials and methods

### Animals

**Fig 1:**
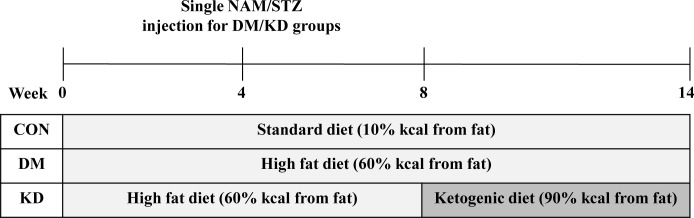
Diagram of experimental procedure. The mice of a ketogenic diet (KD) group were fed a KD from experimental week 8 for 6 weeks. CON, control; DM, diabetes melitus; NAM, nicotinamide; STZ, streptozotocin.

Male C57BL/6J mice (total of 30 mice) of approximately 6-week-old (19~21 g) were obtained from Raon Bio (Yongin, Republic of Korea). The mice were housed 3–4 mice per cage under standard laboratory conditions (a 12-h light/dark cycle, controlled humidity (40 ± 10%), and constant temperature (24 ± 1°C)) with a standard chow diet (10% kcal from fat, D12450J, Research Diets, New Brunswick, NJ, USA) and water provided *ad libitum*. After 1-week of acclimatization, the mice were given either a standard chow diet (CON; n = 10) or a high-fat (HF) chow diet (60% kcal from fat; D12492, Research Diets). A single intraperitoneal injection of streptozotocin (STZ) and nicotinamide (NAM) combined with HF diet feeding was used as a way to induce T2DM. NAM (120 mg/kg, Sigma-Aldrich, St Louis, MO, USA) was injected into the mice assigned to the HF diet at treatment week 4, and it was used to protect the mice from severe pancreatic beta cell damage induced by STZ [29]. STZ (100 mg/kg; Sigma-Aldrich) was dissolved in 0.1 M citric acid buffer (pH 4.4), and injected into the mice after 15 min from NAM injection. After the 8-week treatment period, fasting blood glucose concentrations were measured to confirm whether the diabetic condition was induced, and the cut-off value for the diabetic condition was 200 mg/dL or above of fasting blood glucose. Then, the mice fed with the HF diet were randomly divided into the DM control group (n = 10) and the KD group (n = 10). Then, the HF diet or the KD (90% kcal from fat; D16062902, Research Diets) was provided to the mice according to the assigned diet for the next 6 weeks (Fig 1). Diets were fed *ad libitum*, and the nutritional composition of the three diets is summarized in Table 1. Energy density values were 3.85, 5.24, and 6.7 kcal/g for the standard low-fat diet, the HF diet, and the KD, respectively. The total experimental period was 14 weeks after a 1-week acclimatization period. Body weights and food intake were monitored. After a 14-week experimental period, all mice were anesthetized and euthanized with carbon dioxide after a 12-hour fast [30]. Blood samples were collected by cardiac puncture. Gastrocnemius (GA), soleus, and quadriceps (QU) muscles were dissected, weighed, and collected for analysis. Part of the muscles was stored in 10% formalin solution (Sigma-Aldrich) for histology, and the rest of the muscle tissues was stored at -80°C until use. All protocols and procedures conformed to the specifications outlined in the National Institutes of Health Guiding Principles for the Care and Use of Laboratory Animals to alleviate suffering. It was approved by the Institutional Animal Care and Use Committee at Seoul Women’s University (SWU IACUC 2021A-5).

**Table 1:** Diet composition.

	Standard diet (D12450J)	High-fat diet (D12492)	Ketogenic diet (D16062902)
Contents (g/kg)			
Casein	190	258	165
L-cystine	3	4	2
Corn starch	480	0	0
Maltodextrin 10	118	162	0
Sucrose	65	89	0
Cellulose, BW200	47	65	83
Soybean oil	24	32	41
Lard	19	317	629
Mineral mix S10026	9	13	17
Dicalcium phosphate	12	17	21
Calcium carbonate	5	7	9
Potassium citrate, 1 H_2_O	16	21	27
Vitamin mix V10001	9	13	0
Vitamin mix V10001C	0	0	2
Choline bitartrate	2	3	3
Composition			
Energy (kcal/g)	3.85	5.24	6.7
Protein (% energy)	20	20	10
Carbohydrate (% energy)	70	20	0.1
Fat (% energy)	10	60	89.9

### Oral glucose tolerance test

After overnight fasting for 12 h, an oral glucose tolerance test (OGTT) was performed for all experimental mice. Blood samples were repeatedly obtained from the tail vein. Fasting blood glucose concentrations were measured using the Glucocard 01 Sensor (Arkray, Kyoto, Japan). Then, the mice were challenged with glucose (2 g/kg body weight) dissolved in phosphate-buffered saline via stomach gavage. At 15, 30, 60, 90, and 120 min after the glucose ingestion, glucose concentrations were measured from blood samples obtained from the tail vein. The area under the curve (AUC) was calculated from the glucose concentrations obtained during the OGTT.

### Homeostatic model assessment of insulin resistance

The blood samples were allowed to clot by leaving those at room temperature for 2 hours, then centrifuged at 2,000 × g for 20 min at 4°C. The serum was separated and used for further analyses. Fasting serum insulin was measured using an Ultra Sensitive Mouse Insulin enzyme-linked immunosorbent assay (ELISA) kit (M1104, Morinaga Institute of Biological Science, Inc., Yokohama, Japan), and fasting serum glucose concentrations were analyzed using a glucose assay kit (GAGO20, Sigma-Aldrich). Homeostatic model assessment of insulin resistance (HOMA-IR) was calculated as follows: [fasting glucose concentrations (mmol/L) × fasting insulin concentrations (μU/mL)]/22.5 [31].

### Beta-hydroxybutyrate measurement

Beta-hydroxybutyrate (BHB) concentrations in serum were analyzed using a BHB colorimetric assay kit (K632-100, BioVision, Waltham, MA, USA) according to the manufacturer’s instructions. The samples were incubated with BHB dehydrogenase, and the generated products were reacted with the colorimetric probe. Absorbance was read at 450 nm, and the BHB concentrations were estimated from a standard curve with different amounts of BHB.

### Biochemical measurements

Biochemical measurements were made with commercial assay kits according to the manufacturer’s instructions. Serum concentrations of corticosterone (catalog number: KGE009) and insulin-like growth factor-1 (IGF-1, catalog number: MG100) were analyzed by ELISA kits from R&D systems (Minneapolis, MN, USA). Serum concentrations of albumin (catalog number: 80630) and C-peptide (catalog number: 90050) were measured by ELISA kits from Crystal Chem (Elk Grove Village, IL, USA). And, free fatty acids (FFA, catalog number: MAK044) in serum, pyruvate dehydrogenase (PDH) activity (catalog number: MAK183) and acetyl-CoA (catalog number: MAK039) in GA were quantified by assay kits from Sigma-Aldrich.

### Histology

The hematoxylin and eosin (H&E) staining was used to assess the morphological changes. GA muscle tissues (n = 4 per group) were fixed with a 10% formalin solution, and embedded in paraffin. Paraffin block of GA muscle was sectioned in 4 μm thickness, and stained with H and E. H&E-stained cross-sections were observed and imaged using a LEICA DM750 microscope, provided with an integrated ICC50 camera (LEICA Microsystems, Wetzlar, Germany). ImageJ software (version 1.53t; National Institutes of Health, Bethesda, MD, USA) was used to determine the mean cross-sectional area (CSA) and Minimal Feret’s Diameter of muscle fibers from a representative field (magnification 100 x) of GA tissue for each mouse. Each representative field has more than 100 muscle fibers used for the quantification analyses.

### Grip strength test

A grip strength test was conducted to assess muscle function using a grip strength meter (Bioseb, Pinellas Park, FL, USA). Forelimb, hindlimb, and whole-limb grip strengths were measured. Each test was conducted three times in five sets, with the average recorded in grams per gram of body weight. The test was conducted between 16:00 and 18:00 in a blind setting without knowing the assigned group.

### Gene expression analysis

Total RNA was prepared from about 100 mg of GA muscles using Invitrogen PureLink RNA Mini Kit (12183018A, Thermo Fisher Scientific, Waltham, MA, USA) according to the manufacturer’s instructions. RNA concentrations were determined using a NanoDrop 2000c Spectrophotometer (Thermo Fisher Scientific). cDNA was synthesized using Applied Biosystems High Capacity cDNA Reverse Transcription (RT) Kit (Thermo Fisher Scientific) from 500 ng of total RNA. RT was conducted in 20 μL of total reaction volume, which included random primers, deoxynucleoside triphosphate mix, reverse transcriptase, RT buffer, and 5 μg total RNA with RNase-free water. The RT conditions were 25°C for 10 min, 37°C for 120 min, 85°C for 5 min, and 4°C. Gene expression was analyzed by quantitative PCR using Applied Biosystems Power SYBR Green Master mix (Thermo Fisher Scientific) on a StepOne Plus Real-Time PCR System (Applied Biosystems, Waltham, MA, USA). Primer sequences are listed in Table 2. Relative gene expression was estimated using the comparative Ct method, and 18S was used as the control for sample normalization.

**Table 2:** Primer sequences for real-time reverse transcription polymerase chain reaction.

Gene	5’-Forward-3’	5’-Reverse-3’
*Akt*	TCG TGT GGC AGG ATG TGT AT	ACC TGG TGT CAG TCT CAG AGG
*ASC*	GAG CAG CTG CAA ACG ACT AA	GTC CAC AAA GTG TCC TGT TCT G
*eIF2α*	CAA CGT GGC AGC CTT ACA	TTT CAT GTC ATA AAG TTG TAG GTT AGG
*Foxo1*	CTT CAA GGA TAA GGG CGA CA	GAC AGA TTG TGG CGA ATT GA
*Ire1*	CTG TGG TCA AGA TGG ACT GG	GAA GCG GGA AGT GAA GTA GC
*mTOR*	AGA AGA CAG CGG GGA AGG	GCA TCT TGC CCT GAG GTT C
*NLRP3*	CCC TTG GAG ACA CAG GAC TC	GAG GCT GCA GTT GTC TAA TTC C
*Perk*	AGT CCC TGC TCG AAT CTT CCT	TCC CAA GGC AGA ACA GAT ATA CC
*18S*	GCA ATT ATT CCC CAT GAA CG	GGG ACT TAA TCA ACG CAA GC

*ASC*, apoptosis-associated speck-like protein; *eIF2α*, eukaryotic translation initiation factor 2 *α*; *Foxo1*, forkhead box O1; *Ire1*, inositol-requiring enzyme 1; mTOR, mammalian target of rapamycin; *NLRP3*, nucleotide-binding and oligomerization domain-like receptor family pyrin domain-containing 3; *Perk*, RNA-dependent protein kinase-like ER kinase.

### Statistical analyses

The primary outcome variables of this study are muscle mass, strength, and muscle fiber size; secondary outcome variables are metabolic parameters and gene expression levels of related targets. The sample size was estimated for the detection of a 0.5 mg/g body weight difference in GA muscle between mean values of DM and KD groups at a significance level of 5%, a power of 80%, and a standard deviation of 0.35 mg/g body weight, which was based on findings in a previous study [26]. The sample size was calculated using the epicalc package (V 2.15.1.0) in R software (V 4.2.1). Sufficient power was achieved if 8 mice were included in each group. Data values were expressed as means ± standard error of the mean (SEM) and analyzed on SPSS statistics 26 (IBM Corp, Armonk, NY, USA). Differences in outcome variables among groups were analyzed by one-way analysis of variance and Bonferroni *post-hoc* analysis. Statistical significance was set with *p* < 0.05.

## Results

### Effects of a ketogenic diet on body weight, food intake, and muscle weight

Body weight, weight gain, and food intake (g/day) were greater in the DM group than in the CON group, which was reduced by KD feeding (Table 3). Food intake, expressed as calories per day, was higher in the DM and KD groups compared with the CON group without changes by KD ingestion. Induction of the diabetic condition significantly reduced total muscle mass, especially GA and QU muscles, confirming that muscle mass was decreased in DM. KD feeding increased muscle mass to a similar extent as in the CON group, indicating muscle mass preservation, especially in GA and QU muscles.

**Table 3:** Body weight (BW) changes, food intake, and muscle tissue weights in the ketogenic diet (KD)-fed mice.

	CON	DM	KD
Initial BW (g)	21.15 ± 0.23	21.07 ± 0.32	20.87 ± 0.29
Final BW (g)	33.60 ± 0.71	43.31 ± 1.90 *	32.67 ± 0.51 ^†^
Weight gain (g/day)	0.13 ± 0.01	0.23 ± 0.02 *	0.12 ± 0.00 ^†^
Food intake (g/day)	2.73 ± 0.03	3.02 ± 0.08 *	2.41 ± 0.07 *^,^^†^
Food intake (kcal/day)	10.53 ± 0.13	15.12 ± 0.46 *	14.68 ± 0.54 *
Total muscle mass (% BW)	2.15 ± 0.08	1.50 ± 0.10 *	2.38 ± 0.10 ^†^
Gastrocnemius (% BW)	1.05 ± 0.04	0.78 ± 0.03 *	1.14 ± 0.07 ^†^
Soleus (% BW)	0.04 ± 0.00	0.03 ± 0.00	0.04 ± 0.00
Quadriceps (% BW)	1.07 ± 0.05	0.72 ± 0.06 *	1.19 ± 0.05 ^†^

Data are expressed as means ± SEM (n = 10 per group). One-way analysis of variance with Bonferroni *post-hoc* analysis was used to compare the differences in outcome variables between groups.

* *p* < 0.05 vs. CON group and

^†^ *p* < 0.05 vs. DM group. Total muscle mass included gastrocnemius, soleus, and quadriceps muscles, and was demonstrated as a percentage of BW. CON, control; DM, diabetes.

### A ketogenic diet improved blood glucose homeostasis, and induced nutritional ketosis

The STZ/NAM injection combined with HF diet feeding impaired glucose homeostasis, as shown by higher levels of fasting serum glucose, OGTT AUC, fasting serum insulin, and HOMA-IR (Table 4). KD feeding significantly improved these parameters. In addition, the serum BHB concentration was analyzed to confirm whether KD feeding induced nutritional ketosis. There was no difference in the BHB concentration between CON and DM groups, and KD feeding increased the serum BHB concentration to a level associated with nutritional ketosis (0.5–3.0 mmol/L) [32].

**Table 4:** The effects of a ketogenic diet (KD) on fasting serum glucose, the area under the curve (AUC) of oral glucose tolerance test (OGTT), fasting serum insulin, homeostatic model assessment of insulin resistance (HOMA-IR), and serum beta-hydroxybutyrate (BHB) concentrations in diabetic mice.

	CON	DM	KD
Fasting serum glucose (mg/dL)	91.33 ± 1.98	304.20 ± 4.67 *	174.30 ± 5.68 *^,^^†^
OGTT AUC	14997 ± 908	46206 ± 1452 *	28425 ± 1418 *^,^^†^
Fasting serum insulin (pmol/L)	154.73 ± 10.18	256.76 ± 7.98 *	179.28 ± 4.96 *^,^^†^
HOMA-IR	5.03 ± 0.37	27.79 ± 1.11 *	11.12 ± 0.53 *^,^^†^
Serum BHB (mmol/L)	0.26 ± 0.02	0.24 ± 0.02	1.25 ± 0.29 *^,^^†^

Data are expressed as means ± SEM (n = 10 per group). One-way analysis of variance with Bonferroni *post-hoc* analysis was used to compare the differences in outcome variables between groups.

* *p* < 0.05 vs. CON group and

^†^ *p* < 0.05 vs. DM group. CON, control; DM, diabetes.

**Fig 2:**
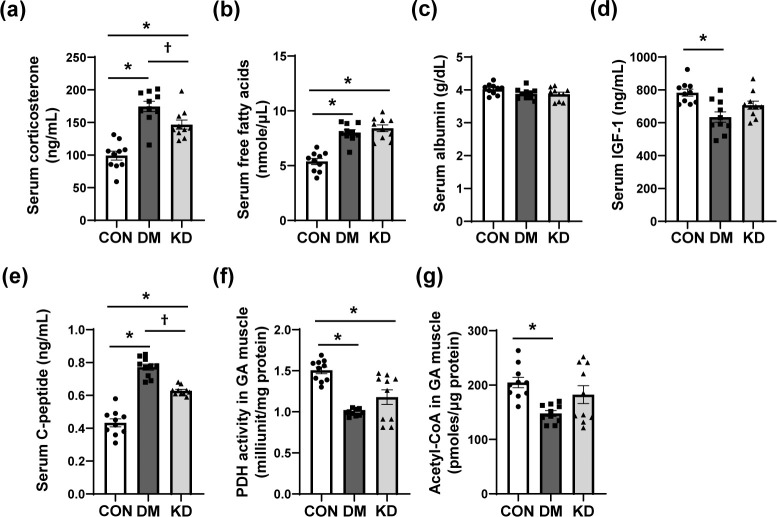
The effects of a ketogenic diet (KD) on serum concentrations of (a) corticosterone, (b) free fatty acids, (c) albumin, (d) insulin-like growth factor-1 (IGF-1), and (e) C-peptide, and pyruvate dehydrogenase (PDH) activity and acetyl-CoA concentration in gastrocnemius (GA) muscles. Data are expressed as means ± SEM (n = 10 per group). One-way analysis of variance with Bonferroni *post-hoc* analysis was used to compare the differences in outcome variables between groups. * *p* < 0.05 vs. CON group and ^†^ *p* < 0.05 vs. DM group. CON, control; DM, diabetes.

Moreover, other related metabolic parameters were analyzed in serum and GA muscles. Serum concentrations of corticosterone, FFA, and C-peptide were increased in DM groups (Fig 2). KD feeding significantly decreased corticosterone and C-peptide in serum without affecting serum FFA. Serum albumin concentrations were similar in all groups. Serum IGF-1 concentrations were reduced in DM groups, and PDH activity and acetyl-CoA concentrations were lowered in GA muscles of DM groups. KD feeding tended to increase PDH activity in GA muscles (*p* = 0.077) without affecting serum IGF-1 and Acetyl-CoA in GA muscles.

### The effects of a ketogenic diet on muscle morphology and cross-sectional area of muscle fiber

**Fig 3:**
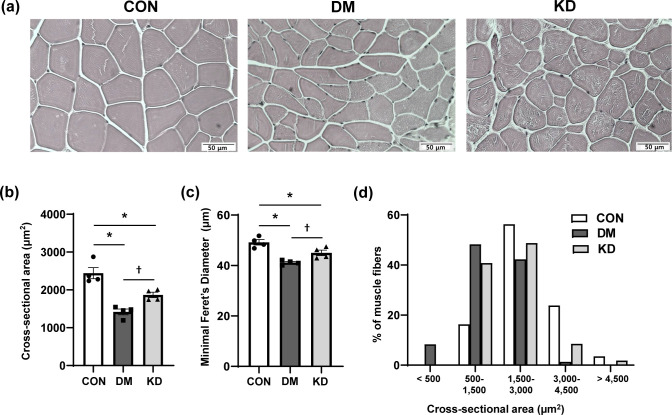
The effects of a ketogenic diet (KD) on (a) gastrocnemius (GA) muscle morphology, (b) cross-sectional areas (CSA) and (c) Minimal Feret’s Diameter of GA muscle fiber, and (d) distribution of CSA of GA muscle fiber. (a) Representative photographs of hematoxylin and eosin (H&E)-stained GA muscle sections. Original magnification, 400x. Scale bar, 50 μm. Data are expressed as means ± SEM (n = 4 per group). One-way analysis of variance with Bonferroni *post-hoc* analysis was used to compare the differences in outcome variables between groups. * *p* < 0.05 vs. CON group and ^†^ *p* < 0.05 vs. DM group. CON, control; DM, diabetes.

**Fig 4:**
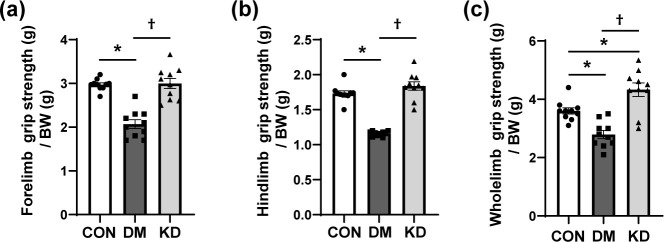
The effects of a ketogenic diet (KD) on muscle strength: (a) forelimb, (b) hindlimb, and (c) wholelimb grip strengths. Data are expressed as means ± SEM (n = 10 per group). One-way analysis of variance with Bonferroni *post-hoc* analysis was used to compare the differences in outcome variables between groups. * *p* < 0.05 vs. CON group and ^†^ *p* < 0.05 vs. DM group. BW, body weight; CON, control; DM, diabetes.

H&E-stained sections of GA muscles were analyzed to investigate the effects of KD feeding on muscle morphology. Muscle fibers were smaller in the DM group, and this decrease was restored by KD feeding (Fig 3A). The CSA and Minimal Feret’s Diameter of GA muscle fibers were quantified to confirm the changes in muscle fiber size (Fig 3B and 3C). The results indicate that the CSA and Minimal Feret’s Diameter of GA muscle fibers were significantly reduced in the DM group compared with the CON group. KD feeding increased the CSA and Minimal Feret’s Diameter of the muscle fibers, suggesting improvements in muscle atrophy. The distribution graph of muscle fiber size showed a higher proportion of larger myofibers in the KD-treated group than in the DM group (Fig 3D). The CSA of muscle fibers in the DM group had the highest distribution in 500 ~ 1,500 μm^2^. The CON and KD groups had the higher proportion of muscle fibers, with CSA of 1,500 ~ 3,000 μm^2^, while the DM group had the lowest proportion in this area range, showing that KD feeding restored muscle atrophy. Forelimb, hindlimb, and wholelimb grip strengths were measured to evaluate muscle function. Muscle strengths were lower in the DM group than in the CON group (Fig 4). KD feeding significantly increased all measured grip strengths compared with the DM group.

### The effects of a ketogenic diet on nucleotide-binding and oligomerization domain-like receptor family pyrin domain-containing 3 (NLRP3) inflammasome- and endoplasmic reticulum stress-related markers in gastrocnemius muscles

**Fig 5:**
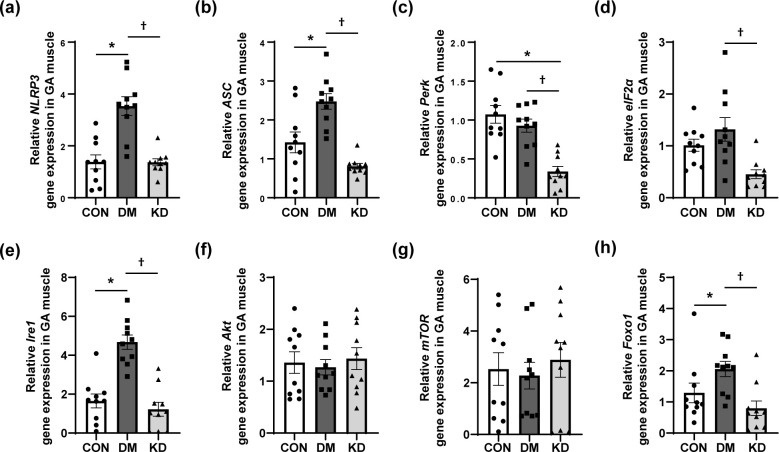
The effects of a ketogenic diet (KD) on nucleotide-binding and oligomerization domain-like receptor family pyrin domain-containing 3 (NLRP3) inflammasome- and endoplasmic reticulum (ER) stress-related markers in gastrocnemius (GA) muscles. Gene expression of (a) NLRP3, (b) apoptosis-associated speck-like protein (ASC), (c) protein kinase RNA-like endoplasmic reticulum kinase (Perk), (d) eukaryotic translation initiation factor 2 alpha (eIF2 alpha), (e) inositol-requiring enzyme 1 (Ire1), (f) Akt, (g) mammalian target of rapamycin (mTOR), and (h) forkhead box O1 (Foxo1) in GA muscles. Data are expressed as means ± SEM (n = 10 per group). One-way analysis of variance with Bonferroni *post-hoc* analysis was used to compare the differences in outcome variables between groups. * *p* < 0.05 vs. CON group and ^†^ *p* < 0.05 vs. DM group. CON, control; DM, diabetes.

NLRP3 inflammasome- and ER stress-related markers were analyzed to investigate whether KD feeding affects the NLRP3 inflammasome and ER stress in GA muscles. Gene expression of the NLRP3 inflammasome complex components NLRP3 and apoptosis-associated speck-like protein (ASC), was upregulated in the GA muscles of the DM group compared with the CON group, and was significantly reduced by KD feeding (Fig 5). Among the analyzed ER stress markers, inositol-requiring enzyme 1 (*Ire1*) was increased in the GA muscles of the DM group compared with the CON group. KD feeding significantly reduced the gene expression of protein kinase RNA-like endoplasmic reticulum kinase (*Perk*), eukaryotic translation initiation factor 2 alpha (*eIF2 alpha*), and *Ire1* in GA muscles (Fig 5). In addition, gene expression of Akt, mammalian target of rapamycin (mTOR) and forkhead box O1 (Foxo1) was analyzed. KD feeding reduced gene expression of Foxo1 in GA muscles (Fig 5H) compared with the DM group without affecting Akt and mTOR (Fig 5F and 5G).

## Discussion

Muscle loss and dysfunction are prevalent in diabetes [2, 3]. Strategies aimed at preventing and treating these complications and their underlying mechanisms need to be investigated. Here, we showed that KD feeding induced nutritional ketosis, with increased BHB synthesis, and improved insulin actions, including glucose control. Moreover, KD feeding preserved muscle mass, muscle fiber size, and muscle function in DM. And, the KD feeding down-regulated the gene expression of NLRP3 inflammasome- and ER stress-related markers, which might be related to the improvements in insulin action and the muscle preservation.

Reduced muscle mass and function were reported in diabetes [20]. Here, a single STZ-NAM injection combined with the HF diet (60% kcal from fat) was utilized to induce the condition of T2DM in mice. The STZ-NAM injection with the HF diet feeding successfully established a rodent model of T2DM with hyperglycemia and hyperinsulinemia, minimizing damage to the pancreas. Moreover, this treatment resulted in reduced muscle mass and function in mice, as shown by lower muscle weights, CSA of muscle fibers, and grip strength.

Studies on the effectiveness of dietary interventions in diabetic sarcopenia are limited; most studies have focused on age-related sarcopenia [33, 34]. Traditional dietary recommendations for sarcopenia include adequate protein intake and supplementation with specific peptides or amino acids [33, 35, 36]. According to a recent systematic review and meta-analysis, indices of specific dietary patterns, including the Alternative Healthy Eating Index and indices of the Mediterranean Diet, were associated with the risk of sarcopenia [37]. However, it needs caution to interpret the association between dietary patterns and sarcopenia due to potential bias.

The KD is a well-known dietary regimen that can reduce symptoms of epilepsy and weight gain [7, 8]. In the current study, body weight gain was significantly lower in KD-fed mice than in the DM group. Food intake (g/day) was also reduced by KD feeding; however, the calorie intake was comparable between DM and KD groups. These confirmed the anti-obesity effects of KD. KD feeding also improved glucose control in DM, as shown by reduced levels of fasting serum glucose, OGTT AUC, fasting serum insulin, and HOMA-IR. These KD-mediated improvements in glucose control are consistent with previous findings in diabetes [11, 12].

Nutritional ketosis can be induced by various dietary modifications, including KD, ketone supplementation, intermittent fasting, or calorie restriction [6], and is considered effective for weight loss, improved cognitive function, and cancer protection [6]. It has an inverse association between the blood ketone concentration and insulin resistance in subjects with T2DM [13], suggesting a relationship between nutritional ketosis and insulin resistance. In the current study, the KD induced nutritional ketosis by increasing the mean BHB concentration to 1.25 mM, and reduced the serum insulin concentration and HOMA-IR, confirming the inverse association between the blood ketone concentration and indices of insulin resistance [13]. In normal conditions, the BHB concentration corresponds to the FFA concentration [38] because BHB is synthesized by subsequent processes: 1) beta-oxidation of fatty acids and 2) ketogenesis from S-acetyl CoA [39]. In diabetic condition, impaired insulin action stimulates lipolysis [40]. Nonetheless, this condition may hinder the uptake and beta-oxidation of fatty acids [40], resulting in the inhibition of subsequent ketogenesis or the suppressed levels of ketone bodies. Therefore, DM mice showed increased serum FFA concentrations without altering serum BHB compared with the lean control group in the current study. Interestingly, KD feeding in DM mice did not affect serum FFA concentrations, but, significantly increased BHB concentrations, suggesting that KD feeding might stimulate the beta-oxidation of fatty acids and subsequent ketogenesis in DM condition.

Here, induction of diabetic condition was accompanied by lower muscle mass, size of muscle fiber, and muscle function, as indicated by reduced grip strength, which was consistent with the previous evidence [2, 3, 19, 41]. Handgrip strength is a simple and reliable measurement of muscle strength and is used to assess muscle function [42]. Lower handgrip strength was reported in subjects with T2DM compared with normoglycemic controls [41]. In mice, a grip strength test was utilized to assess muscle strength [43, 44]. The current study reported reduced muscle mass and grip strength in DM, which was rescued by KD feeding. Collectively, findings of muscle mass and muscle strength in the current study demonstrated the preservative effect of KD on muscle mass and strength.

Impaired insulin signaling, inflammation, and ER stress are thought to underlie the reduction of muscle mass and function in diabetes [19–21]. Defective insulin-mediated protein anabolism leads to a reduction in muscle mass in diabetes, and can be overcome by nutritional intervention, including adequate protein intake [19, 45]. Excessive levels of inflammation are involved with the development and progression of diabetes [22]. The NLRP3 inflammasome is a critical mediator of innate immunity, and is often activated in diabetes [46]. NLRP3 inflammasome was activated by a high concentration of glucose in cells [47], and its activation was up-regulated in patients with T2DM [23]. Activated NLRP3 recruits ASC and caspase-1, producing interleukin-1 beta and inducing the secretion of tumor necrosis factor-alpha and interleukin-6 [46]. Expression of the NLRP3 inflammasome components NLRP3 and ASC, and interleukin-1 beta was higher in subjects with T2DM than in healthy subjects [23]. Furthermore, glucotoxicity induces ER stress, which is associated with reduced insulin action and increased beta-cell death [24]. ER is a cellular organelle that is responsible for the biosynthesis, folding, and assembly of proteins [48]. ER stress occurs when the cellular demands exceed the capacity of ER [48]. For example, increased levels of insulin intermediates and molecules related to insulin signaling can exceed the folding capacity of ER, resulting in ER stress [25]. Subsequently, unfolded or misfolded proteins accumulate in the ER, which initiates unfolded protein response by three ER transmembrane proteins PERK, IRE1 alpha, and activating transcription factor 6 (ATF6) [48]. These three unfolded protein response sensors attempt to increase the ER’s protein folding capacity and to remove misfolded proteins [48]. It is likely that the inability to fold the insulin intermediates and molecules related to insulin signaling and the subsequent elevation of ER stress-related molecules such as PERK, IRE1 alpha, and ATF6 are responsible for the impaired glucose homeostasis in diabetes. Excessive inflammation via NLRP3 inflammasome activation, and ER stress can impair insulin action, which might lead to defects in muscle protein anabolism. In addition to these mechanisms, it is possible that other signaling pathways may be involved with the insulin’s action. Once insulin binds to the insulin receptor, it can activate the phosphoinositide 3-kinase (PI3K)/Akt/mTOR pathway, stimulating protein synthesis [49]. Moreover, activation of the PI3K/Akt pathway inhibits Foxo, which suppresses the transcription of target atrophic genes and protein degradation [50]. In the current study, gene expression of Akt and mTOR was not affected by KD feeding; however, increased Foxo1 expression in DM was reduced by KD feeding. Collectively, KD’s effects on muscles may involve the suppression of protein degradation rather than the stimulation of protein synthesis.

As a potential dietary intervention to improve muscle mass and function in diabetes, the KD was provided to a mouse model of T2DM. KD feeding for 6 weeks significantly improved muscle mass, size of muscle fibers, and grip strength in the current study. These effects were accompanied by improved glucose control and reduced expression of the NLRP3 inflammasome complex components NLRP3 and ASC, and ER stress-related markers Perk, eIF2 alpha, and Ire1. The KD was previously shown to inhibit NLRP3 inflammasome activation in a rat model of osteoarthritis [51]. Another study using a mouse model of ischemic stroke demonstrated that KD feeding reduced NLRP3 inflammasome activation and ER stress in the brain [52]. Few studies have examined the regulatory effects of the KD on NLRP3 inflammasome activation and ER stress in diabetes. Therefore, our results provide new insights into this condition.

Contrasting results exist regarding the effects of KD on skeletal muscle. A study investigating the short-term effect of KD on skeletal muscle and related metabolic parameters reported that a 7-day KD feeding mimicked chronic starvation and resulted in muscle atrophy [53]. Also, the same study reported that a 7-day KD feeding lowered serum concentrations of albumin, glucose, insulin, alanine, and IGF-1, and PDH activity in skeletal muscles, but, increased serum concentrations of FFA and corticosterone [53]. In contrast, a 6-week (42 days) KD feeding preserved muscle mass and strength accompanied by reduced serum corticosterone, insulin, and C-peptide concentrations in the current study. The KD feeding did not alter serum FFA, albumin, and IGF-1, as well as PDH activity and acetyl-CoA in GA muscles. These results suggest that a 6-week KD feeding may suppress the catabolic process and improve insulin resistance in diabetic mice with relative hyperinsulinemia. KD feeding induced rapid decreases in food intake in the initial phase and restored the amount of food intake over time. A possible explanation for the contrasting results among studies regarding the KD-mediated effects on skeletal muscle is that differences in feeding duration and fat contents may influence the KD-mediated effects on skeletal muscle. In our study, the feeding duration was relatively long (6-week feeding of KD), and food intake expressed as calories per day was not altered over a 6-week KD feeding, resulting in the preservative effect of KD on muscle mass and strength.

Regardless of the meaningful findings, the current study has some limitations. First, we assumed that increased synthesis of ketone bodies from the KD led to the beneficial effects of KD on muscle mass and function in DM. However, the direct effects of ketone bodies have not been investigated. Second, the regulatory mechanisms of insulin action are complex, and other mechanisms, beyond NLRP3 inflammasome and ER stress, may underly the KD-mediated effects on muscle. Third, we used a ketogenic diet with little carbohydrate because of the limited availability of commercial ketogenic diets and the need to induce nutritional ketosis. Therefore, future studies should verify the role of ketone bodies on insulin action, muscle mass and function by supplementing ketone bodies, and identify underlying mechanisms for the regulation by ketone body supplementation by utilizing other types of ketogenic diets.

## List of abbreviations

ASC: apoptosis-associated speck-like protein
AUC: area under the curve
BHB: beta-hydroxybutyrate
CSA: cross-sectional area
eIF2α: eukaryotic translation initiation factor 2 alpha
ER: endoplasmic reticulum
Foxo1: forkhead box O1
GA: gastrocnemius
H&E: hematoxylin and eosin
HFD: high-fat diet
HOMA-IR: homeostatic model assessment of insulin resistance
Ire1: inositol-requiring enzyme 1
KD: ketogenic diet
NAM: nicotinamide
NLRP3: nucleotide-binding and oligomerization domain-like receptor family pyrin domain-containing 3
OGTT: oral glucose tolerance test
Perk: RNA-dependent protein kinase-like ER kinase
QU: quadriceps
STZ: streptozotocin
T2DM: type 2 diabetes
